# Determination and controlling of grain structure of metals after laser incidence: Theoretical approach

**DOI:** 10.1038/srep41527

**Published:** 2017-01-30

**Authors:** Amir Reza Ansari Dezfoli, Weng-Sing Hwang, Wei-Chin Huang, Tsung-Wen Tsai

**Affiliations:** 1 Department of Materials Science and Engineering, National Cheng Kung University, No.1, Daxue Rd., East Dist., Tainan City 701, Taiwan; 2 Laser and Additive Manufacturing Technology Center, Industrial Technology Research Institute, Tainan City 701, Taiwan

## Abstract

There are serious questions about the grain structure of metals after laser melting and the ways that it can be controlled. In this regard, the current paper explains the grain structure of metals after laser melting using a new model based on combination of 3D finite element (FE) and cellular automaton (CA) models validated by experimental observation. Competitive grain growth, relation between heat flows and grain orientation and the effect of laser scanning speed on final micro structure are discussed with details. Grains structure after laser melting is founded to be columnar with a tilt angle toward the direction of the laser movement. Furthermore, this investigation shows that the grain orientation is a function of conduction heat flux at molten pool boundary. Moreover, using the secondary laser heat source (SLHS) as a new approach to control the grain structure during the laser melting is presented. The results proved that the grain structure can be controlled and improved significantly using SLHS. Using SLHS, the grain orientation and uniformity can be change easily. In fact, this method can help us to produce materials with different local mechanical properties during laser processing according to their application requirements.

The topic of laser interaction with metals is one of the most interesting topic of present-day physics. Many innovations in metal manufacturing processes like laser welding^1^, laser additive manufacturing^2^,^3^,^4^ and laser surface treatment^5^ is brought by this important phenomena. Laser processing has a number of advantages, such as being easy to apply on complicated 3D surface, having a short process time, using a force free method, being applicable for selective treatment, and causing little distortion due to less thermal penetration^6^,^7^,^8^,^9^.

The molten pool generated by laser heat source can be in “conduction” or “keyhole” modes^10^,^11^,^12^ as a function of laser scanning speed and laser power. In conduction mode, metal is locally melted due to conduction and molten pool can be determined by a shallow and wide shape^10^. In keyhole mode due to high energy intensity a deep and narrow molten pool forms. In this mode, evaporation of atoms and ions leads to forming some small hole in molten pool^12^,^13^. Conduction mode is more popular in laser processing due to high process stability, high quality, better control of the heat input and fewer defect^14^.

Theoretically, laser-metal interaction and laser melting are simulated from different view points. Many researchers have simulated thermal field and thermal stress during laser melting^15^,^16^,^17^,^18^. Micro structure evolution during laser melting is also simulated by different researchers. Phase field modeling is chosen to simulate micro structure during laser melting by some researchers^19^,^20^. Although phase field modelling is an accurate model but this model is suffering from some points such as presently limited to qualitative simulation of a single dendrite or a very small calculation domain due to the large computational capacity needed. In addition, as the complexity of the physical system increases, the phase field modelling needs many assumptions and is not able to capture the behavior of the real physical system. Cellular Automaton (CA) model is also another model which is used for this purpose^21^,^22^. CA model has several advantage like combining the scalability and simplicity. This method also needs lower computational cost in comparison with phase filed modelling. Although very interesting results is achieved by these studies but unfortunately, most of simulations are performed in 2 dimensional. In order to gain more insight about micro structure evolution during laser melting a 3D FE-CA simulation is necessary which may help to solve some limitation in this field. Nowadays, many industries are looking for laser processing methods which can product materials with significantly different mechanical properties at different parts. A common example is manufacturing of porous orthopedics implants^23^. Laser processing is one of the most important processes for manufacturing these implants^24^. The main characteristics for an ideal implants are having good hardness on the surface and having good toughness in their main body. Although by changing laser process parameters such as laser scanning speed, hatch distance, powder layer thickness and laser power some properties may be changed but the feasible parameters are restricted to a small range^25^ and reaching to desires properties during one step manufacturing is almost impossible and then having post treatment is necessary^26^.

Industrial Technology Research Institute (ITRI) from Taiwan has many attempts since 2014 to develop different methods to overcome these limitations during laser processing and find better ways to control the micro structure of metals during laser processing^27^,^28^. This work is also extracted from these efforts which focuses on the investigation of the grain structural evolution of Ti6Al4V after laser incidence. A non-linear transient thermal analysis is performed in order to obtain the temperature field during the single track laser melting. Temperature history of the system is coupled with a 3D-CA model to simulate the grain structure. Moreover, a new approach is presented in this paper called SLHS in order to control solidification process in molten pool. This model allows us to locally control the grain structure of metals during laser processing. The results can help us to gain more understanding about laser-metal interaction from microstructural view point which helps to have more insight about laser processing.

## Results

### Thermal model

**Figure 1:**
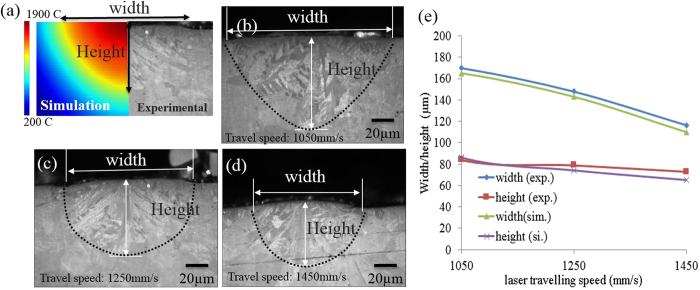
Shape and dimension of the molten pool at the cross-section (YZ plane) after laser incidence. (**a**) Molten pool definition in simulation and experiment. (**b**) Molten pool for laser scanning speed 1050 mm/s. (**c**) Molten pool for laser scanning speed 1250 mm/s. (**c**) Molten pool for laser scanning speed 1450 mm/s. (**e**) Comparison of molten pool dimensions obtained from simulation results and experiment results.

In order to evaluate our thermal simulation, the depth and width of the molten pool are compared with experimental data obtained with laser power of 175W, laser spot size of 35 *μ*m, and laser travel speeds of 1050 mm/s, 1250 mm/s, and 1450 mm/s. This comparison is shown in Fig. 1. The predicted molten pool width and depth are close to the experimental results for all cases. From the results, it is obvious that the melt pool is almost half-cylindrical in shape. The results reveal that by increasing the laser scanning speed, the width and depth of melted area decreases. At a higher laser travelling speed the laser/metal interaction time is naturally lower, and thus less energy is transferred to the surface of the metal.

### Grain structure after laser surface melting

**Figure 2:**
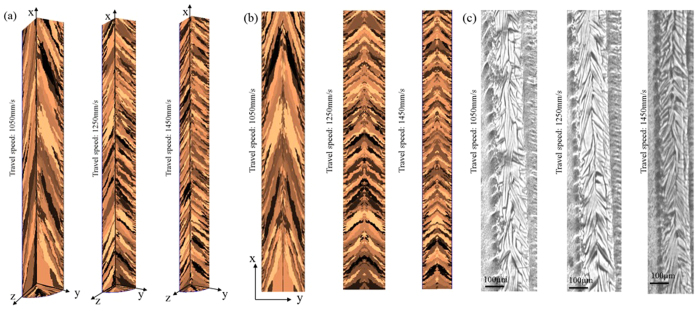
Grain structures of Ti-6Al-4V after laser-metal interaction at various laser scanning speeds. (**a**) 3D simulated grain structure, (**b**) 2D simulated grain structure in XY plane (Top view), (**c**) Experimental grain structure in XY plane.

Using the thermal model and theoretical procedure of CA model presented in this paper, the grain structure of Ti-6Al-4V is simulated and shown in Fig. 2(a and b) for laser power of 175 W, laser spot size of 35 *μ*m, and laser travel speeds of 1050 mm/s, 1250 mm/s, and 1450 mm/s. Furthermore, to evaluate the validity of the present study, the experimental microstructure at XY plane (Top view) is illustrated in Fig. 2c. The same approach is observed for both experimental and simulation. The general grain type for all cases is a directional and columnar grain structure. In fact, during the solidification, the molten pool boarder acts as a set of nucleation sites for new grains, and then grains start to grow toward the laser incidence point. large temperature gradient inside molten pool leads to a high rate of elongated grain grow and thus little chance for new sites to nucleate in the bulk melt during the solidification^29^.

Closer look at molten pool boarder reveals that there is an intense competitive grains growth at the initial stage of solidification. After nucleation, different grains with different preferential growth directions nucleate. Grains with most favorably orientation with respect to the temperature gradient at the solid-liquid interface win this competitive grain growth and less favorably grains are terminated after a short growth close to the molten pool wall. It means that if the favorite orientation of a grain be more parallel to thermal gradient vectors at molten pool boarder, the chance of winning the competitive grain growth is higher. As the results of this competitive grain growth, a lot of small grains can be observed near the molten pool boarder.

The results show that by increasing the laser scanning speed, grains form less parallel to laser direction in the XY and XZ planes. Tilt angle can be defined as the angle between the centerline of grain and laser scanning direction. For laser scanning speed 1050 mm/s, the tilt angle is obtained 35° ± 18° and 20 ± 30° from simulation and experimental study, respectively. These values are 55° ± 8°and 45° ± 10° for laser scanning speed 1250 mm/s and 59° ± 10° and 49° ± 13° for laser scanning speed 1450 mm/s obtained by simulation and experiential studies, respectively.

**Figure 3:**
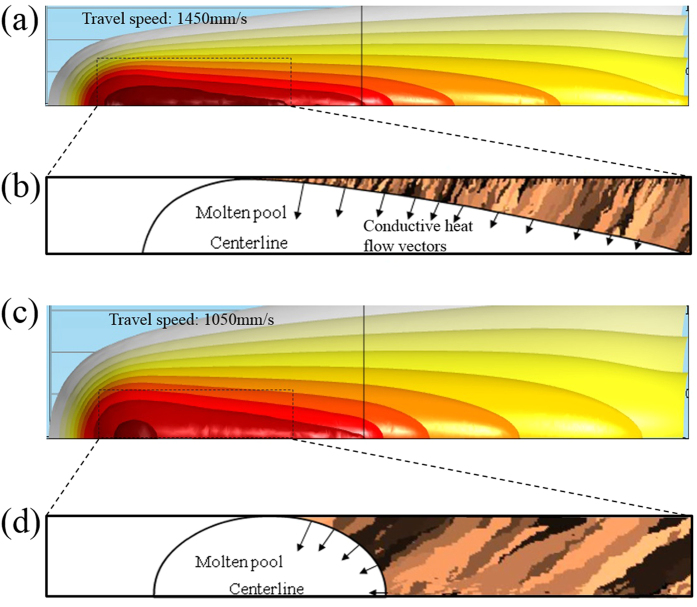
Relation between molten pool shape, heat flow and grain orientation. (**a**) The isothermal surfaces for the XY plane for laser travel speed 1450 mm/s. (**b**) Variation of heat flow vectors near the molten pool for laser scanning speed 1450 mm/s and relation between grains orientation and conductive heat flow vectors inside the molten pool for laser scanning speed 1450 mm/s. (**c**) The isothermal surfaces for the XY plane for laser travel speed 1050 mm/s. (**d**) Variation of heat flow vectors near the molten pool for laser scanning speed 1050 mm/s and relation between grains orientation and conductive heat flow vectors inside the molten pool for laser scanning speed 1050 mm/s.

In order to understand the influence of laser scanning speeds on the grain structure, the molten pool shape and the isothermal surfaces is determined by our thermal model and shown in Fig. 3. The isothermal surfaces are compressed in front of the laser beam and are stretched behind it due to the movement of the laser. The results show that as the laser scanning speed increases, the elliptical shape of the melt pool extends from the laser incidence point. Heat goes out from the melt pool mainly by heat conduction via the bottom of the molten pool. This heat extraction’s pass is the source of solidification and crystallization is mainly controlled by it. The heat flow vectors shown in Fig. 3(b and d) reveals that the conductive heat transfer pass is more parallel with the laser travelling direction at lower laser speed which can be a reason to have grains parallel to laser movement direction at lower laser scanning speed. These less perpendicular heat flow vectors to laser scanning direction at lower scanning speed is due to more round shape of molten pool at lower laser speeds. In fact, the results reveals that the most important phenomena during the laser melting is heat transfer from superheat melt into the bottom cooled solid part.

In conclusion, the molten pool shape is very important because the heat flow is approximately perpendicular to the molten pool boarders where the direction of the heat flow controls the grains orientation during the solidification.

### Controlling the grain structure during laser melting using secondary laser heat source (SLHS)

According to our simulation results, at higher laser scanning speed smaller grain with more tilt angles can be achieved. In the other hand, by decreasing scanning speed, large grains with low tilt angle are appeared inside the molten pool.

Although by changing the laser parameters the grain size and orientation can be changed but some important process properties such as the highest temperature inside molten pool, the dimension of molten pool and even the molten pool mode (conductive and keyhole) can be also changed. In this condition, some problems such as discontinues melt track, formation of defect and pores are happening^27^.

**Figure 4:**
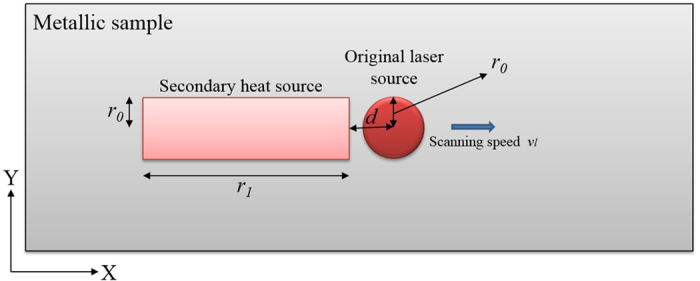
Schematic of using secondary laser heat source (top view) during melting.

In this section using the secondary laser heat source (SLHS) as a grain structure controller is investigated. A schematic of using two different laser heat sources is shown in Fig. 4. The goals are decreasing the temperature gradient inside the molten pool and also changing the shape of molten pool from circular into elliptical at low scanning speed. By decreasing the temperature gradient inside molten pool, more uniform grain structure is formed inside the molten due to having better condition for competitive grain growth and also the shape of molten pool affect the grain orientation strongly as discussed.

**Figure 5:**
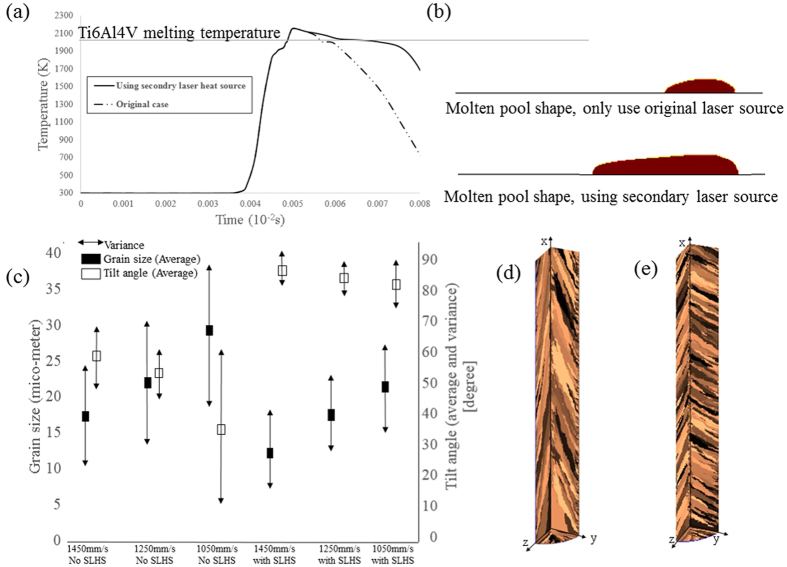
The effect of second laser heat source on grain structure of molten pool. (**a**) Temperature profile. (**b**) Molten pool shape for laser scanning speed 1050 mm/s with and without secondary laser heat source. (**c**) Comparison between grain size and grain orientation for difference condition. (**d**–**e**) Grain structure before and after using secondary laser heat source.

In this model another laser source with following conditions is used:

- Shape of SLHS is rectangular.
- The width of second laser is the same as original laser source (2*r*_*0*_).
- The total laser power of new laser source is the same as the original laser heat source which means heat generated by second laser is much less due to larger area (less energy density).
- Scanning speeds are equal for both lasers.
- Distance between two laser sources (*d*) should be chosen in such a way that the heat from second laser source only decreases the cooling rate and temperature gradient inside molten pool near the melting temperature as shown in Fig. 5a.

The temperature profile and molten pool shape for laser scanning speed 1050 mm/s, *d* = *1.8r*_*0*_ and *r*_*1*_ = *2r*_*0*_ are shown in Fig. 5(a and b). Selecting a proper distance between two heat sources is important. If *d* is chosen too small, the presence of secondary laser heat source can influence the maximum temperature in molten pool and if *d* be too large, the heat coming from secondary laser source only heat up the new solidified microstructure which is useless from grain structure view point. As Fig. 5a shows, in our simulation *d* is considered in that way which the only difference in temperature profile is appeared at the start of solidification where the secondary heat source can control the solidification process. Obviously, in this condition the temperature gradient decreases and moreover the shape on molten pool is change from almost circular to elliptical shape (as shown in Fig. 5b). Less temperature gradient inside molten pool causes better grain competition and more uniform grain structure during the solidification and the elliptical shape causes grains with more tilt angle. In this condition, the grain structure is more uniform and more perpendicular to laser scanning direction. Finally our fantastic achievement can be extracted from Fig. 5c, where the results prove that using SLHS can change the grain orientation almost perpendicular to laser scanning direction and at the same time a high uniform grain structure forms inside the molten pool at lower laser scanning speed. Although the results in Fig. 5c shows for higher laser scanning speed, it is also possible to have grains with perpendicular direction to laser movement direction and smaller size, but using SLHS at lower laser scanning speed makes it possible to have more uniform grain structure and higher tilt angle at a large molten pool. In our study, Grain size for a grain is defined as the diameter of an imaginary sphere which contains the same number of CA cells as original grain. Although by using SLHS and having extra heat power is it expected the grain size increases but due to grain orientation change and less space to grow, grain size decreases.

In addition, the uniformity of grain is defined as function of the grains size variance relating to average grain size as shown in Fig. 5c. Smaller variance for grain size mean more uniformity. Grain structure before and after using SLHS are shown in Fig. 5(d and e).

## Discussion

Grain structure plays an important roles in controlling mechanical properties of metals after laser melting. This study presents a combined model based on the finite element and cellular automaton models, and this is used to establish the grain structure of Ti-6Al-4V during a single laser track melting while the experimental results show that the microstructure inside a scan track is repeated in a similar way for every scan track during laser processing^30^. The numerical results are then compared with the experimental results to verify the approach applied in present study.

Our thermal model reveals that due to moving heat source, the melt pool is not spherical but elongated. The size of melt pool is dependent on the laser scanning speed. It is shown that larger size of molten pool has been obtained at lower scanning speed.

The grains are found to be elongated and columnar after solidification which gives arise to anisotropic properties. The growth direction of elongated grains can be control by heat flow direction which is a function of laser scanning speed. The heat generated by laser is mainly extracted from the melt pool via heat conduction into cooler part of sample. This heat flow properties (size and direction) can control the orientation of elongated grains inside the fusion zone. Moreover, size of elongated grains decreases with increasing the laser travel speed. The results also shows that there is a competitive grain growth at the molten pool boundary which leads to a pronounced preferred orientation of growth. In fact, during this competitive grain growth, grains which preferred orientation (which have higher growth velocity along the heat flow) growth faster and terminate growth of grains with non-preferred orientation.

In the last section, theoretically a new approach proposed in order to control the grain structure during the solidification. In this model, a rectangular secondary laser heat source with the same width is moving behind the original laser source at a certain distance behind it. As discussed the secondary laser heat source can help to elongate molten pool at smaller scanning speeds and also decreases the temperature gradient inside the molten pool. In this condition large molten pool with more uniform/high tilt angle grains can be achieved.

The results showed that grain size, grain orientation and grain boundaries can be changed using SLHS. Most of important mechanical properties of metals such as yield stress, ductility and hardness can be changed by changing these^31^,^32^. So by changing the grain shape (size, orientation and boundaries) some changes are expected in final mechanical properties during laser melting. Due to decreasing grain size after using SLHS in our case (laser melting in single laser track), yield stress and hardness should increase while toughness and uniform elongation are expected to decrease. Thus, during laser processing by turning SLHS off or on, it is possible to produce a metal component with different mechanical properties at different parts.

Some advantages of this new approach (using SLHS) are possibility of changing the grain size and grain orientation at different areas of products to have different mechanical properties at different areas (locally controlling the material microstructure) and reaching to more uniformity in grain structure. In addition, Using SLHS decreases the temperature gradient inside melt (Fig. 5a). It is proved by researchers that less temperature gradient inside the molten pool leads to less surface tension which decreases Marangoni convection where formation of this kind of convection increases the instability of the liquid and increases surface roughness^33^,^34^. Thus, it seems the surface roughness could be also improved using this technique. In addition, obviously less thermal gradient decreases the thermal stress.

Some of limitations of using SLHS are also can be listed as; 1- although the secondary laser heat source can be installed into an original laser heat source package, still some mechanical operations needed to do it. 2- The distance between original and secondary laser heat source should be selected carefully as describes in paper, 3- because SLHS increases the liquid lifetime, the probability of formation of “ball effect” would be higher. 4-such as ordinary laser processing, when using SLHS, process parameters optimization should be done to reach to optimized laser processing parameters such as laser power, scan speed, hatch spacing, layer thickness, scan pattern, etc.

## Methods

### Thermal modelling

The thermal model is simulated using PROCAST software. The incident laser beam is aimed at the surface of the system, with the system considered as bulk Ti-6Al-4V and laser beam moving with the constant velocity of *v*_*l*_. The heat transfer phenomena in the system can be modeled by solving the incompressible Navier–Stokes equations together with the energy equation:

Mass conservation and Momentum equation^35^:


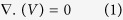



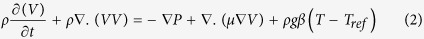


Energy equation^35^:


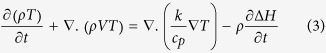


Where *t* is the process time, *c* is the heat capacity, ρ is the density, *k* is the thermal conductivity, *V* is the velocity field, Δ*H* is the latent heat of fusion, *μ* is the viscosity, b is the thermal expansion coefficient and *T* and *T*_*ref*_ are the local and arbitrarily selected reference temperatures.

Initially (before laser incident on the metal surface) the temperature of the system is considered uniformly at room temperature. The most common Gaussian form of the heat source model is used in this study. In this model the laser intensity, *I*, can be defined as refs 15,16:


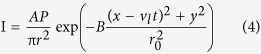


*v*_*l*_ is the laser heat source velocity, *P* is the laser power, *r*_*0*_ is the laser spot radius, *r* is the distance to the laser beam center, *B* is the shape factor of the Gaussian distributed heat flux. *A* is the laser adsorption coefficient, which can be calculated using the reflectively of the material, λ, as *A* = 1 − λ. In order to describe the laser heating and surface cooling due to thermal exchange with the surroundings, following equation is used:


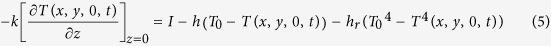


On all other boundaries the following condition is applied:


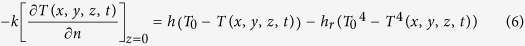


*h* and *hr* are convective heat transfer and radiative heat transfer coefficients. Single track laser melting is a symmetric problem, and therefore symmetry conditions are considered at the center line of the laser heat source:


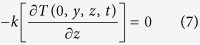


The fluid (melt) velocity at the solid contacts are considered zero. In addition, at the surface of the molten silicon (top), no shear stress condition is considered.

### Cellular Automaton (CA) model

The solid behavior of each CA cell is determined by its own conditions, transformation rules and the condition of its neighbors. This method involves two different phenomena: nucleation and growth. During solidification, some nuclei are first formed (nucleation stage) and then grow to make grains (grain growth).

#### Nucleation

Nucleation can be occurred at the molten pool boarders or inside it. There are thus two types of nucleation sites, and each of these sites become active at a certain critical undercooling temperature. The total density of the nucleation at an undercooled temperature, Δ*T*, can be defined as follows:


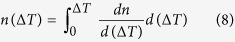


Using the Gaussian distribution, Eq. 8 can be rewritten as


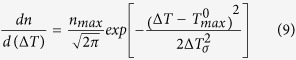


*n*_*max*_ is the maximum nucleation density,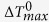 is the mean nucleation undercooling and Δ*T*_*σ*_ is the standard deviation. Undercooling temperature, ΔT, is the difference between melting temperature of titanium alloy, T_melt_, and temperature of a CA cell at undercooled condition. For both nucleation at molten pool wall and inside it, 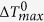, and Δ*T*_*σ*_ are considered 2 °C and 0.5 °C, respectively^36^. The nucleation site density, *n*_*max*_, is strongly dependent on different parameters, such as process conditions and size and number of CA cells (resolution of CA model). Actually the value of *n*_*max*_ should be determined after comparing simulation results with experimental data.

#### Growth model

In this paper due to high thermal gradient during laser melting, the total undercooling temperature is considered as a function of thermal undercooling:


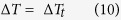


The thermal undercooling can be defined as:


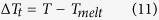


where *T*_*melt*_ is the melting temperature and *T* is the actual temperature in a undercooled-liquid CA cell. The dimensionless thermal undercooling, *Ω*_*t*_, can be define as the thermal undercooling divided by a designated unit thermal undercooling^37^, thus:


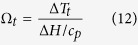


where Δ*H* is the latent heat and *c*_*p*_ is the specific heat. In the other hands, Ivantsov has found a relation for the dimensionless thermal undercooling which is refs 37,38:


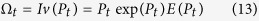


*Iv* is the Ivantsov function, E is the exponential integral function and *P*_*t*_ is the Peclet number defined as:


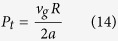


*v*_*g*_ is the interface growth rate, *R* is the tip radius and *a* is the thermal diffusivity. Then total undercooling Δ*T* can be expressed as:


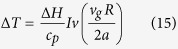


To determine the interface growth rate, one more equation for *v*_*g*_ and *R* is needed. Based on the stability criterion defined by Trivedi^39^ and Kurz and Fisher^40^ another relation will be:


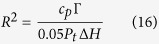


*Γ* is the Gibbs-Thomson coefficient. The growth rate as a function of thermal undercooling can be determine using equations Equations (15) and (16) which is calculated as:


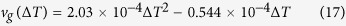


The thermo-physical parameters used to determine *v*_*g*_ are Δ*H* = 370 [kJ/kg], *Γ* = 2 × 10^−7^ [K/m]^41^ and *a* (@ 1650 °C) = 8.9 × 10^−6^ [m^2^/s].

#### FE-CA model procedure

Base on above information about the CA model, we have developed a code to simulate the microstructure of metal after laser incident. The physical domain in 3D space is divided into cells of equal size, 0.1 μm × 0.1 μm × 0.1 μm. Each cell is characterized by three variables: state (solid or liquid), temperature and crystallography orientation. The temperature values are calculated on the macro nodes and it is necessary to be interpolated for use in CA model. Therefore, the following interpolation equation is used:


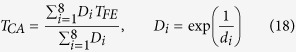


where *d*_*i*_ is the distance between 8 nearest nodes in FE model and center of the CA cell. At the beginning of the simulation, all cells’ states are considered solid and all the crystallographic variables are zero (non-active cell). During the simulation, the temperature of each cell is giving by the thermal simulation. If the temperature of a cell passes the melting temperature, then its state changes to liquid. The grain structure is only calculated for cells which experience melting and solidification (active cell). In addition, the coupling between FE and CA models is done using the temperature recovery method^42^.

The state of a liquid CA cell can be changed into solid again if one of the following conditions happens for it:

- Nucleation
- Capturing by another active-solid CA cell.

In each time step, the density of new nuclei is calculated by Eq. 9 for the molten pool boarders, 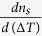, and bulk liquid, 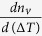. The nucleation densities are then multiplied by the total number of the cells for the molten pool wall and bulk liquid in order to calculate the number of new nucleation sites, therefore:


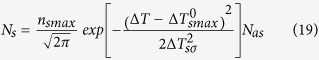



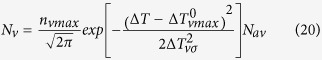


*N*_*s*_ is the number of new nucleation sites at the molten pool wall, *N*_*v*_ is the number of new nucleation sites inside the bulk liquid, *N*_*as*_ is the total number of liquid cells at the molten pool wall, and *N*_*av*_ is total number of liquid cells inside the bulk liquid. After calculating the number of new nucleation sites, a random number (0 < *P*_*i*_ < 1) is assigned to each liquid cell. The nucleation is formed in a cell *i* (located at molten pool wall) if the following condition is satisfied:


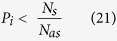


The same rule is applied for cells located inside the bulk liquid. After nucleation at cell *i*, its state is changed from liquid to solid. In addition CA cell *i* should be assigned a preferential growth orientation. A random process is used in order to generate and assign the preferential growth direction. 100 growth orientations are chosen in the 3D spherical space around each nucleated cell as possible growth orientation. After the nucleation, the state of cell *i* is randomly chosen from these 100 orientations. Grains with different orientation grow with different velocity because they have different preferential growth direction. This effect is inserted into our model using he orientation weight coefficient, 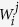. After a certain time, the solidified cell *i* grows enough to capture its nearest neighbor. Assume cell *i* is nucleated and at least one of its 26 neighbors is in liquid state and indicated by index *j*. The growth length of cell *i* with regard to its liquid neighbor *j* at time *t*_*c*_ can be calculated by:


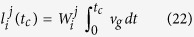



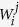
 is the orientation weight coefficient related to the angle between cell *i*’s preferential growth direction and the vector from cell *i* linking to cell *j* (called it vector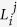). Then orientation weight coefficient, 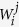 is given by ref. 43:


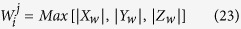


*X*_*w*_, *Y*_*w*_ and *Z*_*w*_ can be calculated by ref. 43:


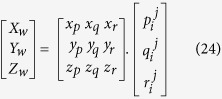


where (*x*_*p*_, *x*_*q*_, *x*_*r*_), (*y*_*p*_, *y*_*q*_, *y*_*r*_) and (*z*_*p*_, *z*_*q*_, *z*_*r*_) are the direction cosines of the [100] and [010] and [001] dendrite arms relating to the coordinate x, y, z axes respectively. (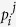, 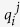, 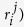 are the direction cosine of the vector 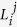 relating to the coordinate x, y, z axes respectively. More information about this approach can be found in Zhu and Hong’s study^43^.

In fact, 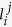 is the growth length between two points *i* and *j (i* has solid state and *j* has liquid state) when we consider the crystallographic orientation of cell *i*^44^. Neighbor *j* is captured by cell *i* if the following condition is satisfied:


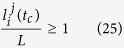


**Figure 6:**
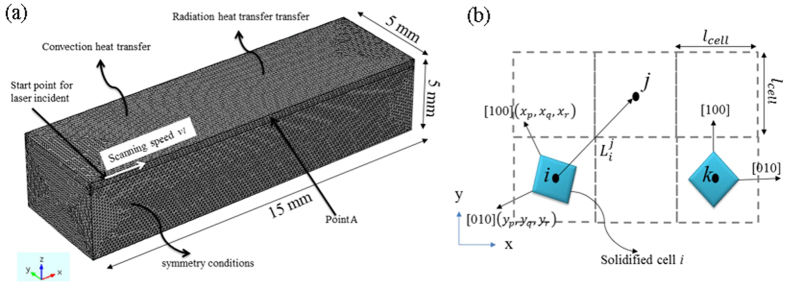
Detail of FE and CA simulations (**a**) Geometry and configuration of the simulated system. (**b**) Some concepts about CA model, two solidified grains *i* and *k* with different favorite orientation, the definition of (*x*_*p*_, *x*_*q*_, *x*_*r*_), (*y*_*p*_, *y*_*q*_, *y*_*r*_) and 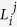 in 2 dimensional.

L = *l*_*cell*_ if *j* is one of the six nearest neighbors, 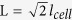 if *j* is one of the twelve second-nearest neighbors, and 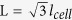 if *j* is one of the eight third-nearest neighbors. If cell *j* be captured by cell *i*, the same grain orientation as cell *i* is assign to cell j. Table 1 summarizes all parameters used in the simulation. In addition the geometry of the system is shown in Fig. 6a. Some basic concepts about CA model is also shown in Fig. 6b. For more clarify Fig. 6b is presented in 2D.

#### Experimental procedure

Ti6Al4V(Ti–6.04Al–4.03V–0.12Fe–0.09O–0.03C–0.009N–23 ppmH) flat plates with a dimension of 10 mm × 10 mm × 5 mm are used in our experiment. Samples surface were cleaned with acetone. Then laser surface melting of Ti6Al4V alloy was performed using a 1250 W continuous wave laser under a gaseous nitrogen atmosphere. The radius of laser spots on titanium sample is considered as 35 μm. Laser operated at the following parameters: power 175 W, scanning rates 1050, 1250 and 1450 mm/s. Treatments were done under an argon protective atmosphere. Optical microstructures were first prepared by mechanical polishing. Polished samples then being etched for optical examination with 1.2 vol% HF, 3.5 vol% HNO_3_ and distilled H_2_O. The microstructure of polished and etched samples were observed by use of an optical microscopy (OM).

## Additional Information

**How to cite this article**: Dezfoli, A. R. A. *et al*. Determination and controlling of grain structure of metals after laser incidence: Theoretical approach. *Sci. Rep.* **7**, 41527; doi: 10.1038/srep41527 (2017).

**Publisher's note:** Springer Nature remains neutral with regard to jurisdictional claims in published maps and institutional affiliations.

## Figures and Tables

**Table 1:** All FE and CA parameters used in the simulation.

Parameter	Value
Laser Power, *P*	175 [W]
Scanning speed, *v*_*l*_	1050/1250/1450 [mm/s]
Laser spot size, *r*_*0*_	35 [μm]
Heat transfer convection, *h*	50 [W/m^2^K]
Heat transfer radiation, *h*_*r*_	1 [W/m^2^K]
Thermal conductivity @ (25, 100, 200, 300, 400, 500, 600, 700, 800, 900, 1100, 1200, 1300, 1400, 1500, 1600, 1650 °C)	7.0, 7.45, 8.75, 10.15, 11.35, 12.6, 14.2, 15.5, 17.8, 20.2, 19.3, 21, 22.9, 23.7, 24.6, 25.8, 27, 28.4 [W/mK]^45^
Specific heat @ (25, 100, 200, 300, 400, 500, 600, 700, 800, 900, 1100, 1200, 1300, 1400, 1500, 1600, 1650 °C)	546, 562, 584, 606, 629, 651, 673, 694, 714, 734, 641, 660, 678, 696, 714, 732, 750, 759 [J/K kg]^45^
Density @ (25, 100, 200, 300, 400, 500, 600, 700, 800, 900, 1100, 1200, 1300, 1400, 1500, 1600, 1650 °C)	4420, 4406, 4395, 4381, 4366, 4350, 4336, 4327, 4309, 4294, 4282, 4267, 4252, 4240, 4225, 4205, 4198, 4189 [kg/m^3^]^45^
Laser adsorption coefficient @ (550, 750, 850, 950, 1000, 1030, 1100, 1300, 1350, T > 1650 °C)	0.40, 0.33, 0.39, 0.30, 0.35, 0.37, 0.27, 0.34, 0.31, 0.1^46^
Viscosity	2.1 × 10^18^exp(−0.0067T) [Kg/(m.s)]^47^
Thermal expansion coefficient	11.0 [10^−6^/K]^48^
T_melting_	1650 [°C]
Latent heat, **ΔH**	370 [kJ/kg]
Gibbs-Thomson coefficient, **Γ**	2 × 10^−7^[K/m]^41^
Nucleation on molten pool wall, **Δ*****T***_***s,max***_**, Δ*****T***_***s,σ***_, ***n***_***s,max***_	2[°C], 0.5[°C], 5 × 10^10^[m^−2^]
Nucleation in liquid, **Δ*****T***_***v,max***_**, Δ*****T***_***v,σ***_, 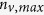	2[°C], 0.5[°C], 5 × 10^14^[m^−3^]
